# Donor *KIR* genotype based outcome prediction after allogeneic stem cell transplantation: no land in sight

**DOI:** 10.3389/fimmu.2024.1350470

**Published:** 2024-04-02

**Authors:** Johannes Schetelig, Henning Baldauf, Falk Heidenreich, Jorinde D. Hoogenboom, Stephen R. Spellman, Alexander Kulagin, Thomas Schroeder, Henrik Sengeloev, Peter Dreger, Edouard Forcade, Jan Vydra, Eva Maria Wagner-Drouet, Goda Choi, Shankara Paneesha, Nuno A. A. Miranda, Alina Tanase, Liesbeth C. de Wreede, Vinzenz Lange, Alexander H. Schmidt, Jürgen Sauter, Joshua A. Fein, Yung-Tsi Bolon, Meilun He, Steven G. E. Marsh, Shahinaz M. Gadalla, Sophie Paczesny, Annalisa Ruggeri, Christian Chabannon, Katharina Fleischhauer

**Affiliations:** ^1^ Department of Internal Medicine I, University Hospital TU Dresden, Dresden, Germany; ^2^ Clinical Trials Unit, DKMS Group, Dresden, Germany; ^3^ EBMT Leiden Study Unit, Leiden, Netherlands; ^4^ Center for International Blood and Marrow Transplant Research, National Marrow Donor Program (NMDP), Minneapolis, MN, United States; ^5^ RM Gorbacheva Research Institute, Pavlov University, St. Petersburg, Russia; ^6^ Klinik für Hämatologie und Stammzelltransplantation, Universitätsklinikum Essen, Essen, Germany; ^7^ Bone Marrow Transplant Unit, Department of Hematology, Rigshospitalet Copenhagen University Hospital, Copenhagen, Denmark; ^8^ Department of Medicine V, University of Heidelberg, Heidelberg, Germany; ^9^ Service Hématologie clinique de Thérapie cellulaire, Centre Hospitalier Universitaire Bordeaux, Université de Bordeaus, Bordeaux, France; ^10^ Transplant Unit and Intensive Care Unit, Institute of Hematology and Bood Transfusion, Prague, Czechia; ^11^ Center for Cellular Immunotherapy and Stem Cell Transplantation, Third Medical Department, Hematology and Oncology, University Cancer Center Mainz, Mainz, Germany; ^12^ University Medical Center Groningen, University of Groningen, Groningen, Netherlands; ^13^ Department of Haematology & Stem Cell Transplantation, Birmingham Heartlands Hospital, Birmingham, United Kingdom; ^14^ Department of Hematology, Instituto Português de Oncologia de Lisboa, Lisboa, Portugal; ^15^ Department of Stem Cell Transplantation, Fundeni Clinical Institute, Bucharest, Romania; ^16^ Biomedical Data Sciences, Leiden University Medical Center, Leiden, Netherlands; ^17^ DKMS Life Science Lab, Dresden, Germany; ^18^ DKMS Group, Tübingen, Germany; ^19^ Department of Hematology & Medical Oncology, Weill Cornell Medicine, New York Presbyterian Hospital, New York, NY, United States; ^20^ Anthony Nolan Research Institute, Royal Free Hospital, London & Cancer Institute, University College London, London, United Kingdom; ^21^ National Cancer Institute, Division of Cancer Epidemiology & Genetics, Bethesda, MD, United States; ^22^ Department of Microbiology and Immunology, Medical University of South Carolina, Charleston, SC, United States; ^23^ Haematology and BMT, Ospedale San Raffaele s.r.l., Milan, Italy; ^24^ Institut Paoli-Calmettes, Centre de Lutte Contre le Cancer, Marseille, France; ^25^ Institute for Experimental Cellular Therapy, University Hospital, Essen, Germany

**Keywords:** killer-cell immunoglobulin-like receptor (KIR), allogeneic hematopoietic cell transplantation (alloHCT), risk of relapse, donor selection, prediction model

## Abstract

Optimizing natural killer (NK) cell alloreactivity could further improve outcome after allogeneic hematopoietic cell transplantation (alloHCT). The donor’s Killer-cell Immunoglobulin-like Receptor (KIR) genotype may provide important information in this regard. In the past decade, different models have been proposed aiming at maximizing NK cell activation by activating KIR-ligand interactions or minimizing inhibitory KIR-ligand interactions. Alternative classifications intended predicting outcome after alloHCT by donor KIR-haplotypes. In the present study, we aimed at validating proposed models and exploring more classification approaches. To this end, we analyzed samples stored at the Collaborative Biobank from HLA-compatible unrelated stem cell donors who had donated for patients with acute myeloid leukemia (AML) or myelodysplastic neoplasm (MDS) and whose outcome data had been reported to EBMT or CIBMTR. The donor *KIR* genotype was determined by high resolution amplicon-based next generation sequencing. We analyzed data from 5,017 transplants. The median patient age at alloHCT was 56 years. Patients were transplanted for AML between 2013 and 2018. Donor-recipient pairs were matched for HLA-A, -B, -C, -DRB1, and -DQB1 (79%) or had single HLA mismatches. Myeloablative conditioning was given to 56% of patients. Fifty-two percent of patients received anti-thymocyte-globulin-based graft-versus-host disease prophylaxis, 32% calcineurin-inhibitor-based prophylaxis, and 7% post-transplant cyclophosphamide-based prophylaxis. We tested several previously reported classifications in multivariable regression analyses but could not confirm outcome associations. Exploratory analyses in 1,939 patients (39%) who were transplanted from donors with homozygous centromeric (cen) or telomeric (tel) *A* or *B* motifs, showed that the donor cen *B/B*-tel *A/A* diplotype was associated with a trend to better event-free survival (HR 0.84, p=.08) and reduced risk of non-relapse mortality (NRM) (HR 0.65, p=.01). When we further dissected the contribution of B subtypes, we found that only the cen *B01/B01*-*telA/A* diplotype was associated with a reduced risk of relapse (HR 0.40, p=.04) while all subtype combinations contributed to a reduced risk of NRM. This exploratory finding has to be validated in an independent data set. In summary, the existing body of evidence is not (yet) consistent enough to recommend use of donor *KIR* genotype information for donor selection in routine clinical practice.

## Introduction

Natural Killer (NK) cells have raised great interest as potential mediators of selective graft-versus-leukemia effects after allogeneic hematopoietic cell transplantation (alloHCT) since the first descriptions of a reduced risk of relapse and improved survival of patients transplanted from haploidentical related donors missing the human leukocyte antigen (HLA) ligands for inhibitory Killer-cell Immunoglobulin-like Receptors (KIR) (1). Characterization of the extensive genetic polymorphism of *KIR* genotypes, including haplotypes with varying numbers of inhibitory and activating *KIR* genes unravelled the increasing complexity of NK cell mediated alloreactivity in alloHCT.

Clinical evidence for NK cell mediated alloreactivity against cancer cells comes from a series of studies demonstrating activity of NK cell transfusions for patients with relapsed or refractory acute myeloid leukaemia (AML) or myelodysplastic neoplasm (MDS) (2–7). Alloreactivity may be triggered by activating receptors and/or inhibitory receptors on the surface of NK cells which bind to classical and non-classical HLA molecules or to non-HLA ligands. KIR play an important role in NK cytotoxicity. While KIR-expression patterns define the NK-cell repertoire phenotype-wise, *KIR* genotypes expose remarkable diversity at an individual and population level. The function of this diversity is poorly understood in health and disease.

Evidence towards a potential role of KIR mediating NK- alloreactivity came from a series of retrospective registry studies on patients with HLA-compatible related or unrelated donors, which showed associations between certain *KIR* genotype patterns of stem cell donors and the risk of relapse after alloHCT (8–13).

The donor’s *KIR* genotype thus may provide critical information and could be utilized for KIR-informed donor selection. In the past decade, different models have been proposed aiming at maximizing NK cell activation through activating KIR encountering their cognate KIR ligands (KIRL) interactions or minimizing repressive signals through inhibitory KIR-KIRL interactions (12–15). Alternative classifications aimed at predicting outcome after alloHCT according to donor *KIR*-haplotypes, thereby integrating information of various sets of encoded activating/inhibitory KIR (16, 17). Another classification approach hypothesized that stronger NK-alloreactivity against leukemic blasts could be triggered in the absence of strong KIR-KIRL interactions with a higher risk of relapse among *KIR2DL2*-positive donors for C1/C1-positive patients, i.e. patients homozygous for the cognate KIR2DL2-ligand in a previous study (18). In the same study, patients heterozygous for the cognate HLA-C ligands transplanted from donors whose genotype did not comprise phylogenetic clade 2 *KIR2DL1* allele, had a higher risk of relapse.

However, it has to be noted that the validation of most donor *KIR* genotype-based prediction models failed so far (19–21). This highlighted the need for independent validation studies with adequate power. Therefore, we set out to validate published models for donor *KIR* genotype-based outcome prediction in a joint EBMT and Center for International Blood and Marrow Transplant Research (CIBMTR) study. Here, we report results from this dataset of approximately 5,000 patients who had received alloHCT for AML or MDS and whose donors had been typed for *KIR* genes at high-resolution.

## Methods

### Inclusion criteria

We conducted a joint study of the EBMT and the CIBMTR. For the study DNA samples from stem cell donors stored at the Collaborative Biobank (www.cobi-biobank.com) were genotyped. 

Patients were included, if they had a first alloHCT from an unrelated donor between January 2013 and December 2018, a diagnosis of AML or MDS and were aged 18 years or more with an available donor sample in the Collaborative Biobank. Patients receiving cord blood transplantation were excluded.

### Medical data used for risk adjustment

Information on the genetic risk and disease stage at transplantation was used to calculate Disease Risk Index (DRI). For this purpose, cytogenetic risk was classified according to the rules for the refined DRI (22) except for chromosome 17p abnormalities which were assigned to the adverse risk group. For patients with missing stage, disease or cytogenetic risk information, DRI group was imputed based on largest frequencies reported in the publication of the refined DRI. The intensity of conditioning regimens was classified according to working definitions of EBMT and CIBMTR (23).

### Sample identity

Donor information was mapped to the medical data of the patient using the Donor ID as a key. Information on the *HLA*-genotype was used to cross-check sample identity by comparing the typing result of the study sample with the original typing results for that donor and by checking HLA-compatibility with the corresponding patient information. HLA compatibility between donors and recipients was assessed based on two-field information for HLA-A, -B, -C, -DRB1 and -DQB1. Donor-recipient pairs, whose HLA-compatibility could not be confirmed, were excluded.

### *KIR* genotyping

Genotyping was performed using a high-resolution short-amplicon-based next generation sequencing workflow. *KIR* typing at the allele-level was based on sequencing of exons 3, 4, 5, 7, 8, and 9 and subsequent bioinformatic analysis as described previously (24).

### Classification of donor *KIR* genotypes

Information on *KIR3DL1* and *KIR2DS1* and their cognate ligands was grouped according to publications by Venstrom et al. (2012) and Boudreau et al. (2017) (12, 13). Further, we classified donors according to *A* versus *B* haplotype motifs using definitions for haplotype assignment as provided by Cooley et al (16, 17). Finally, we calculated scores for selected additive models which integrate information on KIR-KIRL combinations of donor-recipient pairs. We calculated the functional inhibitory KIR count by assigning scores for donor *KIR2DL1*, *KIR2DL2*, *KIR2DL3*, and *KIR3DL1* when the cognate ligands were encoded by the patient HLA genotype as described in the original paper by Boelen et al (25). The inhibitory score was calculated according to the formula in Supplement 1 of the original paper.

Scores integrating information on inhibitory and activating KIR-KIRL interactions were proposed by Krieger et al (14). We calculated the missing KIR-ligand Score, the inhibitory KIR-ligand Score and the activating KIR-ligand Score according to Supplementary Table 3 in the original publication (26). Another model to integrate KIR-KIRL interactions based on unsupervised, systematic testing was proposed by Fein et al. recently (27).

Exploratory analyses focused on individuals with homozygous *KIR* haplotypes to better investigate the effects of certain *KIR* haplotypes. Homozygosity was determined based on haplotypes defined by absence/presence of *KIR* genes. Centromeric and telomeric motifs were classified according to Pyo et al. and Jiang et al (28, 29). Allele-level *KIR* typing results were used to further investigate the allelic composition of homozygous diplotypes. *KIR2DL1* and *KIR2DL3* alleles were assigned to phylogenetic clades according to Hilton et al (30).

### Classification of patient KIR-ligand patterns

HLA-*C* alleles were grouped into C1 and C2 ligands and HLA-*B* alleles were grouped into Bw4-80I/Bw4-80T/Bw6 epitope bearing ligands based on information retrieved from https://www.ebi.ac.uk/ipd/kir/ligand.html (31).

### Statistical analysis

Relapse/progression was the primary endpoint. Event-Free Survival (EFS) and Non-Relapse Mortality (NRM) were secondary endpoints. The study was designed to validate the impact of presence/absence of *KIR2DL1*, *KIR2DL2*, and *KIR2DL3* genes in the donor KIR genotype against combinations of the cognate ligands C1/C2 encoded in the patient *HLA* genotype on the risk of relapse in a stringent confirmatory statistical setting (18). In addition, we tested alternative published models, explored new classification attempts and tested effects in subgroups and major secondary endpoints. No adjustment of the type I error for multiple testing was made for validation tests and exploratory tests.

EFS probabilities were calculated with the Kaplan-Meier estimator and between-group comparisons were performed with the log-rank test. Relapse/progression and NRM were mutually considered as competing risks. For the calculation of cumulative incidences of acute and chronic graft-versus-host disease (GVHD), relapse or death were considered as competing events. Univariable comparisons for these endpoints were performed with the Gray test. All time-to-event endpoints were evaluated in (cause-specific) multivariable Cox proportional hazards regression models. Risk adjustment in the context of multivariable regression models included information on patients’ performance status, age, sex, cytomegalovirus (CMV) serostatus, disease risk index, conditioning intensity, platform for GVHD prophylaxis, HLA-matching, donor age, donor sex, and donor CMV serostatus. Effect sizes were reported as hazard ratios together with 95%-confidence intervals.

## Results

### Patient characteristics

Mapping of data from donors and patients, who met the inclusion criteria, resulted in 5,017 unrelated donor-recipient pairs for whom donor DNA was available for genotyping. The median patient age at alloHCT was 56 years (interquartile range (IQR) 45 years to 64 years). Indications for alloHCT were *de novo* AML for 81.2% of patients, secondary or therapy-associated AML for 6.3% of patients and MDS for 12.5% of patients. Disease risk according to the Disease Risk Index was assessed as high or very high for 28% of patients. Donors and patients were HLA-A, -B, -C, -DRB1, and -DQB1 matched in 78.6% of pairs, one locus mismatched in 20.1% of pairs, and two locus mismatched in 1.3% of pairs. *HLA B* mismatches resulted in KIR ligand changes (Bw4-Bw6) in 1.5% of donor-recipient pairs and *HLA C* mismatches (C1-C2) in 1.6% of donor-recipient pairs. The median donor age was 28 years (IQR, 24 to 36 years). Myeloablative regimens were administered to 57% of the patients and reduced-intensity regimens to 39%. In this cohort of patients with unrelated donors 52% had received ATG for GVHD-prophylaxis and only 7% of patients had received post-transplant cyclophosphamide (PTCY) for GVHD-prophylaxis. Peripheral Blood Stem Cells (PBSC) and Bone Marrow (BM) were used as graft source in 90% and 10% of patients, respectively. Further details on patient characteristics are given in **Table 1**.

**Table 1:** Patient characteristics.

Parameter		Total Cohort	
		N=5017	(100%)
Patient Sex	Male	2736	(55)
	Female	2281	(45)
Age at HCT [years]	Median (IQR, range)	56 (45-64, 18-81)	
Registry	EBMT	3138	(63)
	CIBMTR	1879	(37)
Disease	AML	4075	(81)
	MDS	626	(12)
	sAML/tAML	316	(6)
Disease Risk	Low	170	(3)
	Intermediate	3459	(69)
	High	1308	(26)
	Very High	80	(2)
Karnofsky Status	90-100%	3152	(63)
	80%	1155	(23)
	≤80%	485	(10)
	*Missing information*	225	(4)
HCT-CI	0	1447	(29)
	1-2	1090	(22)
	≥3	1522	(30)
	*Missing information*	958	(19)
GvHD Prophylaxis Platform	ATG based	2589	(52)
	CNI based	1584	(32)
	PTCY based	356	(7)
	*in vivo* alemtuzumab	356	(7)
	*ex vivo* TCD	66	(1)
	*Missing information*	66	(1)
Conditioning Intensity	Myeloablative	2839	(57)
	Reduced	1955	(39)
	Non-myeloablative	109	(2)
	*Missing information*	114	(2)
Conditioning	Chemotherapy-based	4136	(82)
	TBI-based	842	(17)
	*Missing information*	39	(1)
Donor age at HCT [years]	Median (IQR, range)	28 (24-36, 18-61)	
HLA-Match	10/10 matched	3941	(79)
	9/10 (DQB1 mm)	160	(3)
	9/10 (A,B,C or DRB1 mm)	851	(17)
	≤8/10 matched	65	(1)
Patient-Donor Sex Constellation	Male-male	2085	(42)
	Male-female	651	(13)
	Female-male	1490	(30)
	Female-female	791	(16)
Patient-Donor CMV Serostatus	Negative-negative	1403	(28)
	Negative-positive	336	(7)
	Positive-negative	1679	(33)
	Positive-positive	1453	(29)
	*Missing information*	146	(3)
Graft Source	PBSC	4504	(90)
	Bone Marrow	513	(10)
Year of HCT	2013	506	(10)
	2014	990	(20)
	2015	1061	(21)
	2016	933	(19)
	2017	707	(14)
	2018	820	(16)

IQR, interquartile range; EBMT is a brand name (https://www.ebmt.org/); CIBMTR, Center for International Blood and Marrow Transplant Research; AML, acute myeloid leukaemia; sAML, secondary AML; tAML, therapy-related AML; MDS, myelodysplastic neoplasm; HLA, human leukocyte antigen; mm, mismatch; CMV, cytomegalovirus; TBI, total body irradiation; PBSC, peripheral blood stem cells; HCT, hematopoietic cell transplantation.

For the whole cohort, 2-year probabilities were 51% (95%-CI: 49% to 52%) for EFS, 29% (95%-CI: 27% to 30%) for the incidence of relapse/progression, and 20% (95%-CI: 19% to 22%) for NRM. In total, 1,485 relapse events and 1,075 cases of death before observation of relapse or progression were recorded.

### Validation of classifications

First, we attempted to validate associations between selected inhibitory donor *KIR* allele groups, patient KIRL and the risk of relapse which we had observed in an independent previous study (18). Those own findings could not be replicated in this larger dataset. Donor *KIR2DL2* positivity for patients with C1/C1 ligands was not associated with a reduced risk of relapse (HR 1.03, 95%-CI 0.87-1.22, p=.8). And in patients with C1/C2 ligands the presence of *KIR2DL1* alleles belonging to phylogenetic clade 2 in the donor *KIR* genotype was not associated with a reduced risk of relapse (HR 1.1, 95%-CI 0.95-1.28, p=.2).

Next, we attempted to validate alternative donor *KIR* genotype-based prediction models in the current dataset (see **Table 2**). These models included the concept to optimize NK alloreactivity by increasing activating KIR2DS1 activity and limiting KIR3DL1-mediated inhibition (12, 13) and scores which were designed to capture functional inhibitory and activating KIR (14, 25). Very recently, common combinations of activating and inhibitory KIR genes were investigated in a hypothesis-free approach in a CIBMTR data set of AML patients (27). The authors found several associations between distinct genotypes and patient outcomes. In our independent data set, those findings could not be validated. Moreover, we could not validate the impact of donor *KIR* haplotype *B* motifs in the whole cohort and in C1+ recipients (data not shown) (17, 21). Results of donor *KIR* genotype classifications in subgroups of patients defined by conditioning intensity, use of TBI, and patient KIRL (C2/C2 versus C1+) are shown in **Supplementary Tables S2-S7**.

**Table 2:** Model validations for proposed donor *KIR* genotype classifications.

Classifier	N	%	Relapse Incidence		Event-free Survival		Non-relapse Mortality	
			HR (95%-CI)	p	HR (95%-CI)	p	HR (95%-CI)	p
*KIR2DL2* in C1/C1 patients								
***KIR2DL2* absence**	890	48	1		1		1	
***KIR2DL2* presence**	977	52	1.03 (0.87-1.22)	0.8	1.02 (0.89-1.15)	0.8	1.00 (0.82-1.22)	0.98
*KIR2DL1/3* in C1/C2 patients								
***KIR2DL1* clade 2 absence**	984	41	1		1		1	
***KIR2DL1* clade 2 presence**	1414	59	1.10 (0.95-1.28)	0.2	1.13 (1.00-1.27)	0.04	1.16 (0.97-1.39)	0.10
***KIR2DL3* clade 1 absence**	936	40	1		1		1	
***KIR2DL3* clade 1 presence**	1411	60	1.15 (0.98-1.34)	0.08	1.11 (0.98-1.24)	0.09	1.05 (0.88-1.25)	0.6
*KIR3DL1*/HLA-B subtype combinations								
**Strong inhibiting *KIR3DL1***	1279	26	1		1		1	
**Weak-inhibiting *KIR3DL1***	1335	27	1.08 (0.94-1.25)	0.3	1.10 (0.98-1.22)	0.09	1.11 (0.94-1.31)	0.2
**Non-inhibiting *KIR3DL1***	2327	47	1.07 (0.94-1.22)	0.3	1.08 (0.98-1.19)	0.11	1.09 (0.94-1.26)	0.3
*KIR2DS1*/C1/C2 epitope combinations								
***KIR2DS1* neg**	3127	62	1		1		1	
***KIR2DS1* pos/C1+**	1624	32	1.03 (0.92-1.15)	0.6	1.02 (0.93-1.11)	0.7	1.00 (0.88-1.14)	0.96
***KIR2DS1* pos/C2/C2**	266	5	0.83 (0.65-1.06)	0.14	0.82 (0.68-0.99)	0.042	0.81 (0.61-1.08)	0.2
KIR haplotype motif-based classification (16)								
**Cen A/A**	2396	48	1		1		1	
**Cen A/B**	2088	42	1.00 (0.90-1.12)	0.96	0.99 (0.92-1.08)	0.9	0.98 (0.86-1.11)	0.8
**Cen B/B**	491	10	0.86 (0.71-1.04)	0.12	0.89 (0.78-1.03)	0.12	0.94 (0.76-1.15)	0.5
**Tel A/A**	3031	60	1		1		1	
**Tel A/B**	1753	35	1.00 (0.90-1.12)	0.98	0.99 (0.92-1.08)	0.9	0.99 (0.87-1.12)	0.9
**Tel B/B**	233	5	1.01 (0.79-1.30)	0.9	1.01 (0.84-1.22)	0.9	1.02 (0.77-1.36)	0.9
**Neutral (0 or 1 B-motif)**	3495	70	1		1		1	
**Better (≥2 B-motifs, no Cen B/B)**	989	20	1.02 (0.90-1.17)	0.7	1.01 (0.91-1.11)	0.9	0.98 (0.84-1.15)	0.8
**Best (≥2 B-motifs with Cen B/B)**	491	10	0.86 (0.72-1.04)	0.12	0.90 (0.78-1.03)	0.12	0.94 (0.77-1.15)	0.6
Sum inhibitory KIR - Ligands (25)								
**Functional iKIR count (cont.)**	5017	100	1.03 (0.97-1.09)	0.4	1.01 (0.97-1.06)	0.6	1.00 (0.93-1.07)	0.9
**Inhibitory Score (cont.)**	5017	100	1.03 (0.97-1.09)	0.3	1.02 (0.97-1.06)	0.5	1.00 (0.93-1.07)	0.98
Net inhibitory/activating KIR – Ligands (26)								
**Inhibitory (IM-)KIR Score (cont.)**	5017	100	1.02 (0.94-1.12)	0.6	1.02 (0.95-1.09)	0.6	1.01 (0.91-1.12)	0.9
**Weighted (w-)KIR Score (cont.)**	5017	100	1.00 (0.90-1.10)	0.9	0.99 (0.92-1.06)	0.8	0.98 (0.87-1.10)	0.7
**Missing-KIR-Score (cont.)**	5017	100	0.95 (0.90-1.01)	0.11	0.97 (0.92-1.01)	0.11	0.98 (0.92-1.05)	0.6
**Inhibitory-KIR-Score (cont.)**	5017	100	1.05 (1.00-1.10)	0.07	1.04 (1.00-1.08)	0.08	1.02 (0.96-1.08)	0.5
**Activating-KIR-Score (cont.)**	5017	100	0.99 (0.94-1.06)	0.9	0.98 (0.93-1.02)	0.3	0.95 (0.88-1.02)	0.2
Genotype signatures (27)								
**G5 absence**	4644	93	1		1		1	
**G5 presence**	373	7	1.04 (0.86-1.25)	0.7	1.01 (0.87-1.17)	0.9	0.98 (0.78-1.23)	0.8
**G3 absence in Bw4 patients**	2991	95	1		1		1	
**G3 presence in Bw4 patients**	162	5	0.99 (0.73-1.33)	0.9	1.00 (0.80-1.24)	0.97	0.99 (0.71-1.39)	0.98
**G2 absence in C1/C1 patients**	1649	88	1		1		1	
**G2 presence in C1/C1 patients**	218	12	1.04 (0.81-1.35)	0.7	0.97 (0.80-1.18)	0.8	0.87 (0.64-1.20)	0.4

N, number; HR, hazard ratio; p, p-value; neg, negative; pos, positive; cen, centromeric; tel, telomeric; iKIR, inhibitory Killer cell Immunoglobulin like Receptors; cont, continuous; w-KIR-Score, weighted KIR-Score; IM-KIR-Score, inhibitory-missing KIR-ligand Score CIR, cumulative incidence of relapse; Hazard ratios were calculated in (cause-specific) multivariable Cox regression models stratified by registry (CIBMTR or EBMT), and adjusted for patient age, donor age, diagnosis, disease risk index, Karnofsky performance status, conditioning intensity, GvHD prophylaxis, sex match, CMV match, HLA-match, and stem cell source. The p-value of the Wald test is reported.

### Exploratory analyses

#### Investigation of homozygous diplotypes

Taking advantage of the large number of donor-recipient pairs, we sought to investigate the impact of homozygous genotypes. Homozygous genotypes allow for a more direct analysis of the impact of a certain haplotype or haplotype motif because interactions with a divergent haplotype do not have to be considered and the gene dosage is uniform. A total of 4,166 donors were homozygous for either centromeric (cen) or telomeric (tel) KIR motifs and 1,967 donors (39.5% of all donors) were homozygous for both, centromeric and telomeric *KIR* motifs. The distribution of homozygous genotypes classified by *A* or *B* motifs is shown in **Table 3**.

**Table 3:** Frequencies of homozygous centromeric (cen) and/or telomeric (tel) *KIR* motifs.

	tel *A/A*	tel *A/B*	tel *B/B*	Sum
**cen *A/A***	32.5% (1616)	14.3% (709)	1.4% (71)	48.2% (2396)
**cen *A/B***	23.5% (1170)	16.3% (809)	2.2% (109)	42.0% (2088)
**cen *B/B***	4.8% (237)	4.2% (211)	0.9% (43)	9.9% (491)
**Sum**	60.8% (3023)	34.8% (1729)	4.5% (223)	100% (4975*)

*42 genotypes could not be classified according to haplotype A or B motifs due to combinations which did not allow unambiguous assignment.

**Figure 1:**
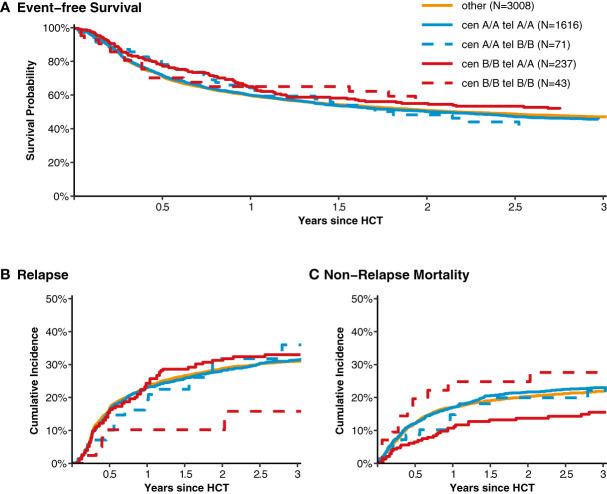
Event-free survival, cumulative incidence of relapse and of non-relapse mortality classified into homozygous versus heterozygous donor *KIR* diplotypes. Panel **(A)** shows event-free survival from transplantation for patients with donors whose genotypes were homozygous for the centromeric (cen) and telomeric (tel) *KIR* gene motifs A/A motifs compared to patients with heterozygous cen & tel *KIR* gene motifs (displayed in orange). Panels **(B, C)** show the cumulative incidences of relapse and non-relapse mortality, respectively.

**Figure 2:**
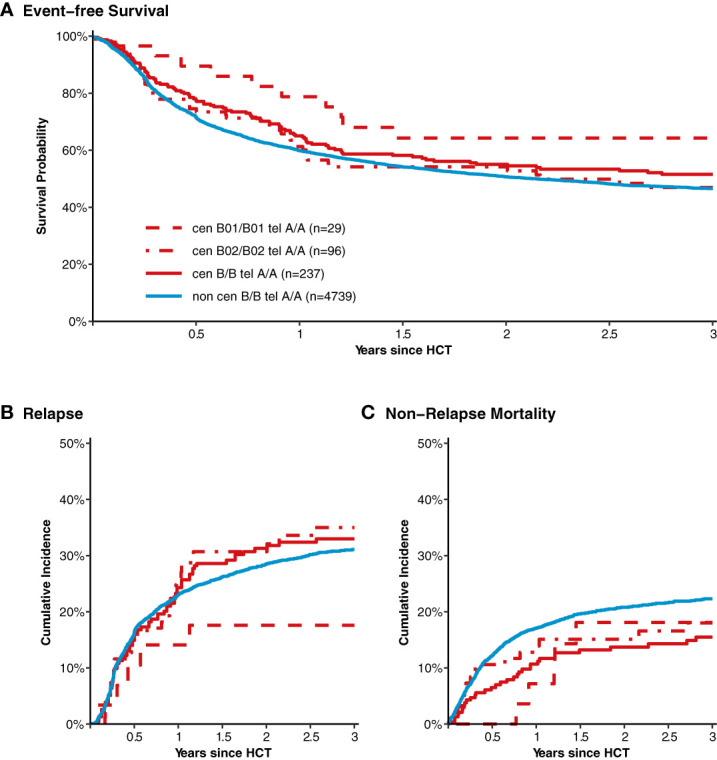
Event-free survival, cumulative incidence of relapse and of non-relapse mortality after transplantation from donors with different homozygous *KIR* diplotypes. Panel **(A)** shows event-free survival of patients after transplantation grouped by the donor *KIR* genotype. Data of patients with segregated homozygous *KIR* donor genotypes (red curves) are compared to all remaining patients (blue curve). High-resolution donor *KIR* genotype *B* motifs were classified into the subgroups *B01* and *B01*. Centromeric *B02* motifs differ from Cen *B01* by deletion of *KIR2DL5*, *KIR2DS3/5*, *KIR2DP1*, and *KIR2DL1* genes. Panels **(B, C)** show corresponding curves for the cumulative incidences of relapse and non-relapse mortality, respectively.

**Figure 3:**
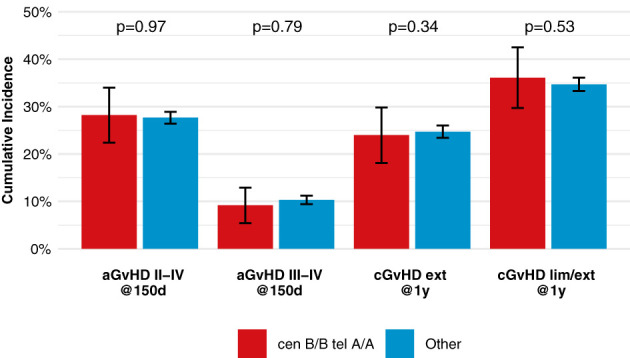
Incidences of acute and chronic GVHD among patients with *cen-B/B tel A/A* donors, shows point estimates of cumulative incidence curves for acute GVHD on day 150 after transplantation and for chronic GVHD at one year after transplantation for patients with *cen-B/B tel A/A* donor KIR genotypes compared to all remaining patients.

After classification of donors with homozygous *KIR* genotype, we compared outcome of donor-recipient pairs with respect to event-free survival, and cumulative incidences of relapse and non-relapse mortality (see **Figure 1**). The small group (N=43) of patients with cen *B/B* tel *B/B KIR* donors showed a trend towards a lower risk of relapse (multivariable Cox regression, HR 0.45, 95%-CI 0.2-1.01; p=.052) but EFS was not different (multivariable Cox regression, HR 0.78, 95%-CI 0.49-1.24; p=.3) compared to patients with non cen *B/B* tel *B/B KIR* donors. Patients who had donors with cen *B/B* tel *A/A KIR* genotypes (N=237) had a trend to better event-free survival (multivariable Cox regression, HR 0.84, 95%-CI 0.70-1.02; p=.08) in multivariate Cox regression modelling compared to donors with non cen *B/B* tel *A/A KIR* genotypes. This trend was caused by a significantly reduced risk of NRM (multivariable Cox regression, HR 0.65, 95%-CI 0.47-0.90; p=.001). The entire multivariable model is presented in **Supplementary Table S1**. Based on this observation, we tried to narrow down the putative beneficial haplotypes by segregating the centromeric *B* motifs of the *cen B/B tel A/A* diplotypes into *B01* and *B02* motifs. *B01* and *B02* motifs differ by absence or presence of *KIR2DL5*, *KIR2DS3*, *KIR2DP1*, and *KIR2DL1* genes. Patients whose donors had *cen B01/B01-telA/A* diplotypes (N=29) showed better EFS (multivariable Cox regression, HR 0.50, 95%-CI 0.27-0.90; p=.02) due to a lower risk for NRM (multivariable Cox regression, HR 0.40, 95%-CI 0.17-0.97; p=.04) compared to non *cen B01/B01-telA/A* donors. We observed no differences with respect to the risk of NRM for the subgroups of cen *B/B* tel *A/A* donors (**Figure 2**). Up to day +150 after alloHCT the cumulative incidence of acute GVHD II-IV and III-IV was 28% (95%-CI: 22% to 34%) and 9% (95%-CI: 5% to 13%) for patients with cen *B/B* tel *A/A* donors compared to 28% (95%-CI: 26% to 29%) and 10% (95%-CI: 9% to 11%) for controls (**Figure 3**). Also, no difference was found for the cumulative incidences of chronic GVHD.

#### Impact of single KIR

In order to complement existing association studies and since many scores integrate information on functional inhibitory KIR, we systematically investigated KIR binding to C1 or C2. We classified high-resolution *KIR* genotypes into phylogenetic clades and tested the impact of the presence versus absence of those KIR with their cognate KIRL. Results of the single KIR – HLA-C-ligand analyses are shown in **Table 4**. Patients with C2/C2 ligands whose donors were *KIR2DL1* phylogenetic clade 3*-*positive (N=190) showed a lower risk of relapse (HR 0.65, 95%-CI 0.46-0.91, p=.012) and better event-free survival (HR 0.65, 95%-CI 0.50-0.84, p=.001). In contrast, patients with C1/C2 ligands whose donors were *KIR2DL1* phylogenetic clade 2*-*positive (N=1,414) showed a trend toward higher NRM (HR 1.16, 95%-CI 0.97-1.39, p=.096) and worse event-free survival (HR 1.13, 95%-CI 1.00-1.27, p=.042). To provide comprehensive results we present the impact of the presence/absence of all KIR in **Supplementary Table S8**.

**Table 4:** Patient outcomes by KIR binding to HLA-C receptors.

Classifier	Ligands	N	%	Relapse Incidence		Event-free Survival		Non-Relapse-Mortality	
				HR (95%-CI)	p	HR (95%-CI)	p	HR (95%-CI)	p
*KIR2DL1* present	C2+	3055	97	1.26 (0.83-1.91)	0.3	1.36 (0.98-1.90)	0.07	1.54 (0.89-2.68)	0.12
absent		95	3	1		1		1	
*KIR2DL2* present	C1+	2197	51	1.00 (0.90-1.12)	1.0	1.00 (0.92-1.09)	0.9	1.00 (0.88-1.14)	0.9
absent		2089	49	1		1		1	
*KIR2DL3* present	C1+	3861	90	1.16 (0.96-1.41)	0.13	1.10 (0.96-1.28)	0.2	1.04 (0.84-1.29)	0.7
absent		425	10	1		1		1	
*KIR2DL1* clade 1 present	C1/C2	1203	50	0.96 (0.82-1.11)	0.6	0.92 (0.82-1.03)	0.2	0.88 (0.74-1.04)	0.14
absent		1195	50	1		1		1	
*KIR2DL1* clade 1 present	C2/C2	345	48	1.14 (0.86-1.51)	0.4	1.21 (0.97-1.50)	0.09	1.28 (0.91-1.79)	0.2
absent		378	52	1		1		1	
*KIR2DL1* clade 2 present	C1/C2	1414	59	1.10 (0.95-1.28)	0.2	1.13 (1.00-1.27)	0.042	1.16 (0.97-1.39)	0.1
absent		984	41	1		1		1	
*KIR2DL1* clade 2 present	C2/C2	439	61	1.00 (0.75-1.33)	0.99	0.97 (0.77-1.21)	0.8	0.94 (0.66-1.33)	0.7
absent		284	39	1		1		1	
*KIR2DL1* clade 3 present	C1/C2	601	25	1.01 (0.85-1.21)	0.9	1.05 (0.92-1.19)	0.5	1.09 (0.90-1.33)	0.4
absent		1797	75	1		1		1	
*KIR2DL1* clade 3 present	C2/C2	190	26	0.65 (0.46-0.91)	0.012	0.65 (0.50-0.84)	0.001	0.66 (0.44-0.99)	0.047
absent		533	74	1		1		1	
*KIR2DL2* present	C1/C2	1220	50	0.98 (0.84-1.14)	0.8	0.99 (0.89-1.11)	0.9	1.02 (0.85-1.21)	0.9
absent		1199	50	1		1		1	
*KIR2DL2* present	C1/C1	977	52	1.03 (0.87-1.22)	0.8	1.02 (0.89-1.15)	0.8	1.00 (0.82-1.22)	0.98
absent		890	48	1		1		1	
*KIR2DL3* clade 1 present	C1/C2	1411	60	1.15 (0.98-1.34)	0.08	1.11 (0.98-1.24)	0.09	1.05 (0.88-1.25)	0.6
absent		936	40	1		1		1	
*KIR2DL3* clade 1 present	C1/C1	1132	63	1.06 (0.88-1.26)	0.6	1.10 (0.96-1.26)	0.2	1.16 (0.94-1.42)	0.2
absent		666	37	1		1		1	
*KIR2DL3* clade 2 present	C1/C2	1133	48	0.97 (0.84-1.13)	0.7	0.93 (0.83-1.04)	0.2	0.87 (0.73-1.04)	0.14
absent		1214	52	1		1		1	
*KIR2DL3* clade 2 present	C1/C1	843	47	1.00 (0.84-1.19)	0.98	0.97 (0.85-1.11)	0.7	0.94 (0.77-1.15)	0.6
absent		955	53	1		1		1	

## Discussion

In reference to studies where donor NK-cell transfusions from haploidentical relatives induced remissions of patients with AML, NK alloreactivity is thought to contribute to graft-versus leukemia effects after alloHCT (3, 5–7, 32, 33). Yet, no consistent model exists which allows for the prediction of NK alloreactivity for HLA-matched and –mismatched transplantation. We here present the largest study investigating different classifications for donor *KIR*-genotype based outcome prediction, so far.

In the confirmatory part of the study, we aimed at validating associations between certain *KIR* genotypes and patient outcomes, which we had observed in an independent data set (19). The disruption of inhibitory signals for NK cells by down-regulation of KIR ligands from the surface of leukemic blasts is at the core of many predictive models (34, 35). Building on the assumption that phylogenetic clades share different strength of KIR – KIRL binding affinities, we classified *KIR2DL1* and *KIR2DL3* alleles into their respective clades and showed that certain phylogenetic clades were associated with the risk of relapse in patients with C1/C2-ligand combinations (18, 30). However, the findings of our previous study could not be confirmed in this independent study (see **Tables 2**, **4**). Alternative explanations for the discordant findings could be uncharacterized differences between the patient cohorts resulting in differential impact of NK alloreactivity or that the previous finding was incidental. In an extended set of analyses in the present study (see **Table 4**), where we investigated systematically all KIR with HLA-C ligands, one association stood out: Patients with C2/C2 ligands whose donors were *KIR2DL1* phylogenetic clade 3*-*positive (N=190) showed a lower risk of relapse (HR 0.65, 95%-CI 0.46-0.91, p=.012). This association should be validated in an independent study.

Further, we investigated homozygous *KIR* genotypes building on previous work to define KIR haplotypes (36). Our motivation for this approach was that with less complexity in the setting of homozygosity, signals could be more easily detected. Furthermore, this approach appeared as a logical extension of *KIR* genotype classifications building on *KIR* haplotype motifs (16, 21, 37). Notably, we did not find significant associations between *cen B/B* motifs and a reduced risk of relapse. However, patients with donors who were homozygous for the *cen B/tel A* haplotype had a trend to better EFS (HR 0.84, p=.08) and lower NRM (HR 0.65, p=.01). The lower risk of NRM could not be linked to a reduced risk of GVHD. Further investigating the role of the *B01* and *B02* subtypes showed a reduced risk of relapse only for patients with homozygous *cen B01/tel A* genotypes. Since the centromeric *B01* motif contains *KIR2DL1* clade 3 alleles, this finding partly reflects the association reported above (30, 38). Yet, it has to be noted that the results are not in line with a smaller study of 890 donor-recipient pairs which reported that *B02* protects better against relapse than *B01* (37). Moreover, Weisdorf et al. reported that *cen B* motifs were associated with a reduced risk of relapse in C1/C1 or C1/C2 patients but not in C2/C2 patients (21). We did not observe this subset effect in exploratory analyses (**Supplementary Tables S6, 7**). However, since Weisdorf et al. did not distinguish between *B01* and *B02* motifs for their analysis, the impact cannot be assigned to specific centromeric *B* motifs.

We also attempted to validate published models for outcome prediction after mostly matched unrelated donor transplantation (see **Table 2**). None of the classifications predicted the risk of relapse and EFS significantly as originally published. We found a lower risk of relapse among patients who had received reduced intensity or non-myeloablative conditioning and had donors with two centromeric *B* motifs (**Supplementary Table S3**). However, this subgroup effect was not observed in a previous study on 1,140 patients with MDS or secondary AML, who had received reduced-intensity or non-myeloablative conditioning [Supplementary Table S2A of Schetelig et al., 2021 (20)]. So, this finding should be interpreted with caution.

Given the disappointing validation studies, it may help to reflect on the specific challenges in this research field. First, small animal models to explore human NK alloreactivity after transplantation, do not exist and cytotoxicity assays do not mimic the complex process of NK cell education. Yet, the plasticity of NK cells is remarkable and averts autoimmunity reliably. As an example, until now, it is not clear if in the setting of alloHCT functional inhibitory KIR, i.e. KIR who encounter their cognate ligand, exert more powerful anti-leukemic effects than non-functional KIR. Further, still not all KIR-ligands are known. Second, the diversity of *KIR* genotypes is substantial. With the availability of high-resolution *KIR* genotyping the number of alleles increased substantially and, as of now, more than 1,600 distinct *KIR* alleles have been described (24, 31). When combined with compound KIR-ligands (C1, C2, Bw4 and Bw6), hundreds of possibilities may be tested. Ambiguity in some genotype calls further hampers optimal statistical analysis of the effect of KIR haplotypes on outcomes. Recently, an expectation maximization-based algorithm has been proposed to take these ambiguous values properly into account in the analysis, leading to improved estimates of haplotype frequencies. However, even then the complexity of the KIR infrastructure makes obtaining unbiased effect estimates very difficult (39). This problem is further aggravated since it is unclear, if NK alloreactivity interacts with conditioning intensity, the use of certain drugs or total-body irradiation, the type of GVHD-prophylaxis, or the graft source (21). The poor understanding of human NK cell biology spawned multiple classifications and hypotheses on patient subsets more susceptible to NK allo reactivity. This setting constitutes a multiplicity problem which is hard to control. Finally, the extent to which peptides displayed in the HLA groove modify KIR–binding has been elicited only rudimentarily, but could be a major force which determines graft-versus-leukemia effects (40, 41). As a consequence, we believe that prediction models have to build on rigorous confirmatory testing in independent, adequately powered studies. Whether artificial intelligence could help resolving the problem, is unknown. While artificial intelligence is a powerful tool to create complex classifications when abundant data is available it does not resolve the multiplicity problem when data are scarce.

In three independent studies, we analysed data on 8,943 patients with AML or MDS whose donors had been typed for KIR genes at the allele-level (19, 20). These data can be accessed for validation studies of other research groups. However, future large studies will be critical. The current shift to post transplant cyclophosphamide (PTCY)-based GVHD-prophylaxis might have an important impact on NK alloreactivity, since cyclophosphamide eliminates most mature donor NK cells infused with the graft (42). Further studies are thus warranted to unravel PTCY-associated changes of NK alloreactivity after alloHCT.

In summary, despite the availability of *KIR* genotype information for more than 3 million stem cell donors, no donor *KIR*-genotype based algorithm for unrelated donor selection can be recommended for clinical practice. It is still not possible to predict patient outcome based on donor *KIR* genotype information at present.

## Data availability statement

The dataset of this study may be accessed by academic research groups beginning 12 months and ending 48 months following article publication. Access is conditional on approval by the responsible registries and signed data transfer agreements. Three organizations are responsible for the data, the Cellular Therapy & Immunobiology Working Party (CTIWP) of EBMT, the Immunobiology Working Committee of the Center for International Blood and Marrow Transplant Research (CIBMTR), and the Collaborative Biobank (www.cobi-biobank.com).

## Ethics statement

All stem cell donors had provided written informed consent when they contributed a sample to the biobank. Donor information was linked to patient outcome data with a unique donor identifier. The study was approved by the Ethical Committee at the Technische Universtität (TU) Dresden, Germany, the Cellular Therapy and Immunobiology Working Party of EBMT, and the Institutional Review Board of the National Marrow Donor Program (NMDP; on behalf of CIBMTR).

## Author contributions

JSc: Writing – original draft, Conceptualization, Investigation, Methodology, Project administration, Writing – review & editing. HB: Writing – original draft, Conceptualization, Data curation, Formal Analysis, Investigation, Methodology, Software, Visualization, Writing – review & editing. FH: Writing – review & editing, Conceptualization, Investigation, Methodology, Supervision. JH: Writing – review & editing, Data curation. SS: Writing – review & editing, Conceptualization, Data curation, Investigation, Methodology, Project administration, Supervision. AK: Writing – review & editing, Data curation, Investigation. TS: Writing – review & editing, Data curation, Investigation. HS: Writing – review & editing, Data curation, Investigation. PD: Writing – review & editing, Data curation, Investigation. EF: Writing – review & editing, Data curation, Investigation. JV: Writing – review & editing, Data curation, Investigation. EW-D: Writing – review & editing, Data curation, Investigation. GC: Writing – review & editing, Data curation, Investigation. ShP: Writing – review & editing, Data curation, Investigation. NM: Writing – review & editing, Data curation, Investigation. AT: Writing – review & editing, Data curation, Investigation. LW: Writing – review & editing, Data curation, Investigation, Methodology, Supervision. VL: Writing – review & editing, Conceptualization, Data curation, Formal Analysis, Investigation, Methodology, Project administration, Supervision. AS: Writing – review & editing, Conceptualization, Investigation, Project administration, Resources, Supervision. JSa: Writing – review & editing, Conceptualization, Data curation, Investigation, Methodology, Supervision. JF: Writing – review & editing, Conceptualization, Investigation, Methodology. Y-TB: Writing – review & editing, Data curation, Investigation. MH: Writing – review & editing, Data curation, Investigation. SM: Writing – review & editing, Conceptualization, Investigation, Supervision. SG: Writing – review & editing, Conceptualization, Investigation, Supervision. SoP: Writing – review & editing, Conceptualization, Investigation, Supervision. AR: Writing – review & editing, Conceptualization, Investigation, Supervision. CC: Writing – review & editing, Conceptualization, Investigation, Supervision. KF: Writing – review & editing, Conceptualization, Data curation, Investigation, Methodology, Supervision.
