# Supplementary material for: Donor KIR genotype based outcome prediction after allogeneic stem cell transplantation: no land in sight
Source: Front Immunol. 2024 Apr 2;15:1350470. doi: 10.3389/fimmu.2024.1350470 (PMC11019434; doi:10.3389/fimmu.2024.1350470)
Supplement: Supplementary file 1 [file DataSheet_1.pdf]

Supplementary Material for

DONOR KIR GENOTYPE BASED OUTCOME PREDICTION AFTER ALLOGENEIC STEM CELL TRANSPLANTATION: NO LAND IN SIGHT!

Johannes Schetelig[1,2*], Henning Baldauf[2], Falk Heidenreich[1,2], Jorinde D. Hoogenboom[3], Stephen R. Spellman[4], Aleksander Kulagin[5], Thomas Schroeder[6], Henrik Sengeloev[7], Peter Dreger[8], Edouard Forcade[9], Jan Vydra[10], Eva Maria Wagner-Drouet[11], Goda Choi[12], Shankara Paneesha[13], Nuno Miranda[14], Alina Tanase[15], Liesbeth C. de Wreede[16], Vinzenz Lange[17], Alexander H. Schmidt[17,18], Jürgen Sauter[18], Joshua A Fein[19], Yung-Tsi Bolon[4], Meilun He[4], Steven GE Marsh[20], Shahinaz M Gadalla[21], Sophie Paczesny[22], Annalisa Ruggeri[23], Christian Chabannon[24], Katharina Fleischhauer[25]

on behalf of the Cellular Therapy & Immunobiology Working Party of the European Society for Blood and Marrow Transplantation (EBMT) and the Immunobiology Working Committee of the Center for International Blood and Marrow Transplant Research (CIBMTR)

Table of Content

Table S1. Multivariable Cox regression model with haplotype classifier..3

Table S2. Donor KIR genotype classifications in myeloablative transplants 5

Table S3. Donor KIR genotype classifications in reduced intensity/non-myeloablative transplants 6

Table S4. Donor KIR genotype classifications in TBI-based transplantation...................................7

Table S5. Donor KIR genotype classifications in non-TBI-based conditioning...............................8

Table S6. Donor KIR genotype classifications for C1+ patients 9

Table S7. Donor KIR genotype classifications for C2/C2 patients...10

Table S8. Patient outcomes by presence of KIR genes 11

Figure S1. Event-free survival, cumulative incidence of relapse and non-relapse mortality after transplantation from donors with homozygous centromeric or telomeric KIR gene motifs...........12

Table S1. Multivariable Cox regression model with haplotype classifier

**Variable**		**Overall Survival**		**Relapse Incidence**		**Event-Free Survival**		**Non-Relapse Mortality**	
		HR (95%-CI)	p	HR (95%-CI)	p	HR (95%-CI)	p	HR (95%-CI)	p
Haplotypes	non cen B/B tel A/A (N=4738)	1		1		1		1	
	cen B/B – tel A/A (N=237)	0.85 (0.69-1.04)	0.11	0.99 (0.78-1.25)	0.9	0.84 (0.70-1.02)	0.08	0.65 (0.47-0.90)	0.01
Patient age	(per 10 years)	1.16 (1.12-1.21)	<.001	1.02 (0.97-1.06)	0.5	1.11 (1.07-1.15)	<.001	1.27 (1.20-1.35)	<.001
Donor age	(per 10 years)	1.04 (0.99-1.09)	0.09	0.94 (0.89-1.00)	0.04	1.01 (0.97-1.06)	0.5	1.12 (1.05-1.19)	0.001
Diagnosis	AML	1		1		1		1	
	sAML/tAML	1.23 (1.05-1.45)	0.01	1.22 (1.00-1.49)	0.05	1.20 (1.03-1.40)	0.02	1.16 (0.91-1.48)	0.2
	MDS/ MDS/MPN	0.83 (0.72-0.95)	0.006	0.72 (0.60-0.86)	<.001	0.86 (0.76-0.98)	0.02	1.05 (0.87-1.27)	0.6
Disease Risk Index	low	1		1		1		1	
	intermediate	1.18 (0.90-1.56)	0.2	1.26 (0.91-1.75)	0.2	1.18 (0.92-1.51)	0.2	1.06 (0.72-1.57)	0.8
	high	2.15 (1.62-2.86)	<.001	2.56 (1.83-3.58)	<.001	2.21 (1.71-2.86)	<.001	1.79 (1.20-2.68)	0.004
	very high	3.08 (2.11-4.48)	<.001	4.32 (2.80-6.66)	<.001	2.98 (2.09-4.25)	<.001	1.44 (0.75-2.77)	0.3
Karnofsky performance status	90 or 100	1		1		1		1	
	80	1.12 (1.02-1.24)	0.02	1.05 (0.93-1.19)	0.4	1.12 (1.02-1.23)	0.02	1.23 (1.06-1.43)	0.005
	<80	1.32 (1.15-1.51)	<.001	1.11 (0.93-1.33)	0.2	1.28 (1.12-1.46)	<.001	1.53 (1.26-1.85)	<.001
	missing information	1.24 (1.01-1.53)	0.04	1.11 (0.85-1.45)	0.4	1.15 (0.94-1.41)	0.2	1.22 (0.90-1.66)	0.2
Conditioning intensity	Myeloablative	1		1		1		1	
	Reduced/ Non-myeloablative	1.02 (0.93-1.12)	0.7	1.04 (0.92-1.16)	0.5	1.04 (0.95-1.13)	0.4	1.03 (0.90-1.18)	0.6
	missing information	1.07 (0.81-1.41)	0.6	1.06 (0.75-1.50)	0.7	1.07 (0.82-1.38)	0.6	1.08 (0.73-1.62)	0.7
GvHD prophylaxis platform	ATG based	1		1		1		1	
	CNI based*	1.03 (0.93-1.14)	0.6	0.96 (0.84-1.09)	0.5	0.99 (0.90-1.09)	0.9	1.04 (0.89-1.20)	0.6
	PtCy based	0.75 (0.62-0.90)	0.002	0.91 (0.74-1.12)	0.4	0.80 (0.68-0.95)	0.012	0.66 (0.50-0.88)	0.005
	alemtuzumab based	1.15 (0.98-1.35)	0.1	1.17 (0.96-1.43)	0.12	1.13 (0.97-1.32)	0.11	1.08 (0.86-1.37)	0.5
	ex vivo TCD	0.82 (0.55-1.23)	0.3	1.20 (0.79-1.82)	0.4	0.92 (0.64-1.33)	0.7	0.48 (0.21-1.07)	0.07
Sex match (patient/donor)	male/male	1		1		1		1	
	male/female	1.11 (0.98-1.26)	0.09	0.93 (0.79-1.10)	0.4	1.11 (0.98-1.25)	0.09	1.35 (1.14-1.60)	0.001
	female/male	0.94 (0.85-1.04)	0.2	1.03 (0.91-1.16)	0.7	0.99 (0.90-1.08)	0.8	0.93 (0.80-1.08)	0.4
	female/female	0.99 (0.87-1.12)	0.9	1.09 (0.94-1.27)	0.3	1.07 (0.95-1.20)	0.3	1.04 (0.86-1.25)	0.7
CMV match (patient/donor)	negative/negative	1		1		1		1	
	negative/positive	1.17 (0.98-1.39)	0.08	1.11 (0.89-1.37)	0.4	1.15 (0.98-1.36)	0.09	1.22 (0.95-1.58)	0.12

	positive/negative	1.16 (1.04-1.29)	0.008	0.96 (0.84-1.09)	0.5	1.07 (0.96-1.18)	0.2	1.25 (1.06-1.47)	0.007
	positive/positive	1.26 (1.12-1.40)	<.001	1.06 (0.93-1.22)	0.4	1.17 (1.05-1.30)	0.004	1.35 (1.14-1.59)	<.001
	missing information	1.39 (1.06-1.81)	0.02	0.96 (0.67-1.39)	0.8	1.32 (1.02-1.69)	0.03	1.93 (1.36-2.73)	<.001
HLA-match	10/10	1		1		1		1	
	≤ 9/10	1.34 (1.21-1.48)	<.001	1.16 (1.02-1.32)	0.02	1.28 (1.16-1.41)	<.001	1.45 (1.26-1.67)	<.001
Stem cell source	Peripheral Blood Stem Cells	1		1		1		1	
	Bone Marrow	1.00 (0.87-1.15)	0.98	0.95 (0.80-1.13)	0.6	0.98 (0.86-1.12)	0.7	1.02 (0.83-1.25)	0.9

Legend: cen, centromeric; tel, telomeric; AML, acute myeloid leukemia; sAML secondary AML; tAML therapy-related AML; MDS, myelodysplastic neoplasia; MPN, myeloproliferative neoplasia; CNI, calcineurin-inhibitor; TCD, t-cell depletion; * 66 patients with missing details were assigned to this group; PtCy, post-transplant cyclophosphamide; CMV, cytomegalovirus; HLA, human leukocyte antigen;

Table S2. Donor KIR genotype classifications in myeloablative transplants

Classifier	N	(%)	Relapse Incidence HR (95%-CI)	p	Event-free Survival HR (95%-CI)	p	Non-Relapse Mortality HR (95%-CI)	p
***KIR2DL2* in C1/C1 patients**								
KIR2DL2 absence	524	(51)	1		1		1	
KIR2DL2 presence	513	(49)	1.10 (0.87- 1.39)	0.4	1.04 (0.87-1.25)	0.6	0.98 (0.74-1.31)	0.9
***KIR2DL1/3* in C1/C2 patients**								
KIR2DL1 clade 2 absence	524	(39)	1		1		1	
KIR2DL1 clade 2 presence	836	(61)	1.18 (0.96-1.45)	0.12	1.09 (0.93-1.28)	0.3	0.98 (0.77-1.26)	0.9
KIR2DL3 clade 1 absence	507	(38)	1		1		1	
KIR2DL3 clade 1 presence	820	(62)	1.22 (0.99-1.51)	0.06	1.08 (0.92-1.26)	0.4	0.89 (0.70-1.15)	0.4
***KIR3DL1*/HLA-B subtype combinations**								
Strong inhibiting *KIR3DL1*	719	(26)	1		1		1	
Weak-inhibiting *KIR3DL1*	753	(27)	1.09 (0.90-1.32)	0.4	1.12 (0.97-1.31)	0.12	1.18 (0.93-1.49)	0.2
Non-inhibiting *KIR3DL1*	1317	(47)	1.07 (0.90-1.28)	0.4	1.07 (0.93-1.22)	0.3	1.06 (0.85-1.31)	0.6
***KIR2DS1*/C1C2 epitope combinations**								
KIR2DS1 neg	1773	(62)	1		1		1	
KIR2DS1 pos / C1+	914	(32)	1.08 (0.93-1.26)	0.3	1.07 (0.96-1.20)	0.2	1.06 (0.89-1.27)	0.5
KIR2DS1 pos / C2/C2	152	(5)	0.84 (0.61-1.17)	0.3	0.86 (0.67-1.11)	0.2	0.91 (0.61-1.34)	0.6
**KIR haplotype motif-based classification**								
Cen A/A	1377	(49)	1		1		1	
Cen A/B	1149	(41)	1.00 (0.87-1.16)	0.97	0.96 (0.86-1.08)	0.5	0.90 (0.75-1.08)	0.3
Cen B/B	285	(10)	1.01 (0.80-1.29)	0.9	0.99 (0.82-1.19)	0.9	0.95 (0.71-1.27)	0.7
Tel A/A	1711	(60)	1		1		1	
Tel A/B	1004	(35)	1.02 (0.88-1.18)	0.8	1.02 (0.91-1.14)	0.7	1.02 (0.85-1.21)	0.9
Tel B/B	124	(4)	1.18 (0.85-1.63)	0.3	1.20 (0.94-1.54)	0.14	1.25 (0.85-1.85)	0.3
Neutral (0 or 1 B-motif)	1969	(70)	1		1		1	
Better (≥2 B-motifs, no Cen B/B)	557	(20)	1.13 (0.95-1.34)	0.2	1.06 (0.93-1.22)	0.4	0.96 (0.77-1.20)	0.7
Best (≥2 B-motifs with Cen B/B)	285	(10)	1.04 (0.82-1.32)	0.7	1.02 (0.85-1.22)	0.8	0.99 (0.74-1.31)	0.9
**Sum inhibitory KIR - Ligands**								
Functional iKIR count (cont.)	2839	(100)	1.05 (0.97-1.13)	0.3	1.04 (0.98-1.10)	0.2	1.02 (0.93-1.13)	0.6
Inhibitory Score (cont.)	2839	(100)	1.06 (0.98-1.15)	0.2	1.05 (0.98-1.12)	0.14	1.03 (0.94-1.14)	0.5
**Net inhibitory / activating KIR – Ligands**								
Inhibitory (IM-)KIR Score (cont.)	2839	(100)	0.98 (0.87-1.11)	0.8	0.96 (0.87-1.05)	0.4	0.92 (0.79-1.06)	0.2
Weighted (w-)KIR Score (cont.)	2839	(100)	0.95 (0.84-1.09)	0.5	0.92 (0.83-1.02)	0.12	0.88 (0.75-1.03)	0.12
Missing-KIR-Score (cont.)	2839	(100)	0.92 (0.85-0.99)	0.023	0.93 (0.87-0.98)	0.011	0.94 (0.86-1.04)	0.2
Inhibitory-KIR-Score (cont.)	2839	(100)	1.07 (1.00-1.15)	0.054	1.05 (1.00-1.11)	0.08	1.02 (0.94-1.11)	0.7
Activating-KIR-Score (cont.)	2839	(100)	1.05 (0.97-1.13)	0.2	1.02 (0.96-1.08)	0.6	0.97 (0.88-1.07)	0.5
**Genotype signatures**								
G5 absence	2626	(92)	1		1		1	
G5 presence	213	(8)	0.98 (0.75-1.27)	0.9	0.93 (0.76-1.14)	0.5	0.87 (0.63-1.21)	0.4
G3 absence in Bw4 patients	1679	(95)	1		1		1	
G3 presence in Bw4 patients	95	(5)	1.15 (0.79-1.69)	0.5	1.07 (0.80-1.44)	0.7	0.96 (0.60-1.54)	0.9
G2 absence in C1/C1 patients	920	(89)	1		1		1	
G2 presence in C1/C1 patients	117	(11)	1.15 (0.81- 1.64)	0.4	1.13 (0.86-1.48)	0.4	1.08 (0.70-1.66)	0.7

Legend: N, number; HR, hazard ratio; p, p-value; neg, negative; pos, positive; cen, centromeric; tel, telomeric; iKIR, inhibitory Killer cell Immunoglobulin like Receptors; cont, continuous; w-KIR-Score, weighted KIR-Score; IM-KIR-Score, inhibitory-missing KIR-ligand Score CIR, cumulative incidence of relapse; Hazard ratios were calculated in (cause-specific) multivariable Cox regression models stratified by registry (CIBMTR or EBMT), and adjusted for patient age, donor age, diagnosis, disease risk index, Karnofsky performance status, GvHD prophylaxis, sex match, CMV match, HLA-match, and stem cell source. The p-value of the Wald test is reported.

Table S3. Donor KIR genotype classifications in reduced intensity/non-myeloablative transplants

Classifier	N	(%)	Relapse Incidence HR (95%-CI)	p	Event-free Survival HR (95%-CI)	p	Non-Relapse Mortality HR (95%-CI)	p
***KIR2DL2* in C1/C1 patients**								
KIR2DL2 absence	349	(44)	1		1		1	
KIR2DL2 presence	440	(56)	0.91 (0.71- 1.17)	0.5	0.99 (0.82- 1.20)	0.9	1.11 (0.83- 1.48)	0.5
***KIR2DL1/3* in C1/C2 patients**								
KIR2DL1 clade 2 absence	439	(45)	1		1		1	
KIR2DL1 clade 2 presence	541	(55)	0.97 (0.76- 1.23)	0.8	1.14 (0.96-1.37)	0.14	1.40 (1.07-1.83)	0.014
KIR2DL3 clade 1 absence	411	(43)	1		1		1	
KIR2DL3 clade 1 presence	553	(57)	1.03 (0.81- 1.31)	0.8	1.12 (0.94-1.34)	0.2	1.25 (0.95-1.64)	0.11
***KIR3DL1*/HLA-B subtype combinations**								
Strong inhibiting *KIR3DL1*	532	(26)	1		1		1	
Weak-inhibiting *KIR3DL1*	563	(28)	1.02 (0.82- 1.27)	0.8	1.04 (0.88-1.22)	0.7	1.04 (0.82-1.33)	0.7
Non-inhibiting *KIR3DL1*	946	(46)	1.03 (0.85- 1.26)	0.7	1.08 (0.93-1.25)	0.3	1.12 (0.90-1.39)	0.3
***KIR2DS1*/C1C2 epitope combinations**								
KIR2DS1 neg	1280	(62)	1		1		1	
KIR2DS1 pos / C1+	676	(33)	0.95 (0.80- 1.13)	0.5	0.95 (0.84-1.08)	0.4	0.96 (0.79-1.16)	0.7
KIR2DS1 pos / C2/C2	108	(5)	0.76 (0.51- 1.14)	0.2	0.79 (0.59-1.06)	0.11	0.82 (0.53-1.26)	0.4
**KIR haplotype motif-based classification**								
Cen A/A	961	(47)	1		1		1	
Cen A/B	892	(43)	0.96 (0.81- 1.13)	0.6	1.02 (0.90-1.15)	0.7	1.11 (0.92-1.34)	0.3
Cen B/B	200	(10)	0.69 (0.51- 0.94)	0.019	0.79 (0.63-0.98)	0.033	0.92 (0.67-1.25)	0.6
Tel A/A	1251	(61)	1		1		1	
Tel A/B	711	(34)	0.95 (0.80- 1.13)	0.6	0.96 (0.85-1.09)	0.6	0.98 (0.81-1.19)	0.8
Tel B/B	102	(5)	0.89 (0.61- 1.30)	0.5	0.85 (0.64-1.13)	0.3	0.82 (0.54-1.26)	0.4
Neutral (0 or 1 B-motif)	1446	(70)	1		1		1	
Better (≥2 B-motifs, no Cen B/B)	407	(20)	0.87 (0.71- 1.07)	0.2	0.94 (0.80-1.09)	0.4	1.02 (0.82-1.28)	0.9
Best (≥2 B-motifs with Cen B/B)	200	(10)	0.69 (0.51- 0.93)	0.014	0.77 (0.62-0.95)	0.016	0.88 (0.65-1.19)	0.4
**Sum inhibitory KIR - Ligands**								
Functional iKIR count (cont.)	2064	(100)	1.00 (0.91- 1.09)	0.9	0.99 (0.92-1.06)	0.7	0.98 (0.88-1.08)	0.7
Inhibitory Score (cont.)	2064	(100)	0.99 (0.90- 1.09)	0.9	0.98 (0.92-1.05)	0.6	0.98 (0.88-1.08)	0.6
**Net inhibitory / activating KIR – Ligands**								
Inhibitory (IM-)KIR Score (cont.)	2064	(100)	1.04 (0.91- 1.18)	0.6	1.09 (0.98-1.20)	0.10	1.16 (1.00-1.35)	0.053
Weighted (w-)KIR Score (cont.)	2064	(100)	1.00 (0.86- 1.16)	0.97	1.06 (0.95-1.19)	0.3	1.16 (0.98-1.37)	0.09
Missing-KIR-Score (cont.)	2064	(100)	0.99 (0.90- 1.08)	0.8	1.00 (0.94-1.07)	0.9	1.03 (0.93-1.14)	0.6
Inhibitory-KIR-Score (cont.)	2064	(100)	1.02 (0.95- 1.11)	0.6	1.03 (0.97-1.09)	0.4	1.03 (0.94-1.13)	0.5
Activating-KIR-Score (cont.)	2064	(100)	0.91 (0.82- 1.01)	0.07	0.93 (0.86-1.00)	0.054	0.95 (0.85-1.06)	0.4
**Genotype signatures**								
G5 absence	1914	(93)	1		1		1	
G5 presence	150	(7)	1.10 (0.81- 1.49)	0.5	1.13 (0.91-1.42)	0.3	1.19 (0.86-1.65)	0.3
G3 absence in Bw4 patients	1257	(95)	1		1		1	
G3 presence in Bw4 patients	63	(5)	0.67 (0.39- 1.15)	0.14	0.89 (0.62-1.28)	0.5	1.18 (0.72-1.94)	0.5
G2 absence in C1/C1 patients	692	(88)	1		1		1	
G2 presence in C1/C1 patients	97	(12)	1.06 (0.73- 1.55)	0.8	0.88 (0.66- 1.19)	0.4	0.70 (0.44- 1.13)	0.14

Legend: N, number; HR, hazard ratio; p, p-value; neg, negative; pos, positive; cen, centromeric; tel, telomeric; iKIR, inhibitory Killer cell Immunoglobulin like Receptors; cont, continuous; w-KIR-Score, weighted KIR-Score; IM-KIR-Score, inhibitory-missing KIR-ligand Score CIR, cumulative incidence of relapse; Hazard ratios were calculated in (cause-specific) multivariable Cox regression models stratified by registry (CIBMTR or EBMT), and adjusted for patient age, donor age, diagnosis, disease risk index, Karnofsky performance status, GvHD prophylaxis, sex match, CMV match, HLA-match, and stem cell source. The p-value of the Wald test is reported.

Table S4. Donor KIR genotype classifications in TBI-based transplantation

Classifier	N	(%)	Relapse Incidence HR (95%-CI)	p	Event-free Survival HR (95%-CI)	p	Non-Relapse Mortality HR (95%-CI)	p
***KIR2DL2* in C1/C1 patients**								
KIR2DL2 absence	156	(46)	1		1		1	
KIR2DL2 presence	185	(54)	0.85 (0.56-1.31)	0.5	0.83 (0.60- 1.14)	0.2	0.83 (0.50- 1.38)	0.5
***KIR2DL1/3* in C1/C2 patients**								
KIR2DL1 clade 2 absence	145	(39)	1		1		1	
KIR2DL1 clade 2 presence	229	(61)	1.12 (0.75-1.67)	0.6	1.25 (0.92-1.70)	0.2	1.37 (0.85-2.22)	0.2
KIR2DL3 clade 1 absence	142	(39)	1		1		1	
KIR2DL3 clade 1 presence	222	(61)	1.22 (0.81- 1.85)	0.3	1.25 (0.92-1.71)	0.2	1.22 (0.76-1.97)	0.4
***KIR3DL1*/HLA-B subtype combinations**								
Strong inhibiting *KIR3DL1*	192	(23)	1		1		1	
Weak-inhibiting *KIR3DL1*	229	(28)	0.92 (0.65- 1.32)	0.7	1.19 (0.90-1.57)	0.2	1.72 (1.10-2.69)	0.018
Non-inhibiting *KIR3DL1*	409	(49)	0.86 (0.63- 1.19)	0.4	1.06 (0.82-1.36)	0.7	1.43 (0.94-2.18)	0.09
***KIR2DS1*/C1C2 epitope combinations**								
KIR2DS1 neg	532	(63)	1		1		1	
KIR2DS1 pos / C1+	269	(32)	0.99 (0.75- 1.30)	0.95	1.05 (0.85-1.29)	0.6	1.16 (0.84-1.59)	0.4
KIR2DS1 pos / C2/C2	41	(5)	0.89 (0.48- 1.66)	0.7	0.72 (0.43-1.20)	0.2	0.50 (0.20-1.26)	0.14
**KIR haplotype motif-based classification**								
Cen A/A	402	(48)	1		1		1	
Cen A/B	357	(43)	0.98 (0.75- 1.27)	0.9	0.87 (0.71-1.07)	0.2	0.72 (0.52-1.01)	0.054
Cen B/B	78	(9)	0.78 (0.47- 1.30)	0.3	0.90 (0.64-1.29)	0.6	1.05 (0.64-1.73)	0.8
Tel A/A	513	(61)	1		1		1	
Tel A/B	296	(35)	0.96 (0.73- 1.25)	0.7	1.01 (0.83-1.24)	0.9	1.11 (0.81-1.52)	0.5
Tel B/B	33	(4)	1.17 (0.64- 2.14)	0.6	0.94 (0.56-1.57)	0.8	0.61 (0.22-1.68)	0.3
Neutral (0 or 1 B-motif)	588	(70)	1		1		1	
Better (≥2 B-motifs, no Cen B/B)	171	(20)	1.00 (0.73- 1.37)	1.0	0.92 (0.72-1.18)	0.5	0.81 (0.54-1.21)	0.3
Best (≥2 B-motifs with Cen B/B)	78	(9)	0.79 (0.48- 1.30)	0.4	0.95 (0.67-1.34)	0.8	1.17 (0.72-1.89)	0.5
**Sum inhibitory KIR - Ligands**								
Functional iKIR count (cont.)	842	(100)	1.03 (0.89- 1.20)	0.7	0.98 (0.88-1.10)	0.7	0.92 (0.77-1.09)	0.3
Inhibitory Score (cont.)	842	(100)	1.03 (0.89- 1.20)	0.7	0.99 (0.88-1.11)	0.8	0.93 (0.78-1.11)	0.4
**Net inhibitory / activating KIR – Ligands**								
Inhibitory (IM-)KIR Score (cont.)	842	(100)	1.00 (0.80- 1.26)	1.0	0.92 (0.78-1.09)	0.3	0.81 (0.62-1.05)	0.12
Weighted (w-)KIR Score (cont.)	842	(100)	0.98 (0.77- 1.26)	0.9	0.90 (0.75-1.09)	0.3	0.78 (0.58-1.05)	0.10
Missing-KIR-Score (cont.)	842	(100)	0.99 (0.87- 1.13)	0.9	0.98 (0.89-1.09)	0.8	0.97 (0.82-1.15)	0.7
Inhibitory-KIR-Score (cont.)	842	(100)	1.01 (0.89- 1.14)	0.9	0.99 (0.90-1.09)	0.8	0.96 (0.83-1.11)	0.6
Activating-KIR-Score (cont.)	842	(100)	0.97 (0.83- 1.13)	0.7	0.96 (0.86-1.08)	0.5	0.95 (0.80-1.13)	0.6
**Genotype signatures**								
G5 absence	778	(92)	1		1		1	
G5 presence	64	(8)	0.89 (0.54- 1.48)	0.7	1.04 (0.72-1.48)	0.8	1.24 (0.74-2.08)	0.4
G3 absence in Bw4 patients	466	(94)	1		1		1	
G3 presence in Bw4 patients	29	(6)	0.94 (0.47-1.84)	0.8	0.76 (0.44- 1.32)	0.3	0.50 (0.19-1.30)	0.2
G2 absence in C1/C1 patients	303	(89)	1		1		1	
G2 presence in C1/C1 patients	38	(11)	1.09 (0.57-2.09)	0.8	1.32 (0.82- 2.12)	0.3	1.69 (0.82- 3.49)	0.2

Legend: TBI, total body irradiation; N, number; HR, hazard ratio; p, p-value; neg, negative; pos, positive; cen, centromeric; tel, telomeric; iKIR, inhibitory Killer cell Immunoglobulin like Receptors; cont, continuous; w-KIR-Score, weighted KIR-Score; IM-KIR-Score, inhibitory-missing KIR-ligand Score CIR, cumulative incidence of relapse; Hazard ratios were calculated in (cause-specific) multivariable Cox regression models stratified by registry (CIBMTR or EBMT), and adjusted for patient age, donor age, diagnosis, disease risk index, Karnofsky performance status, conditioning intensity, GvHD prophylaxis, sex match, CMV match, HLA-match, and stem cell source. The p-value of the Wald test is reported.

Table S5. Donor KIR genotype classifications in non-TBI-based conditioning

Classifier	N	(%)	Relapse Incidence		Event-free Survival		Non-Relapse Mortality	
			HR (95%-CI)	p	HR (95%-CI)	p	HR (95%-CI)	p
***KIR2DL2* in C1/C1 patients**								
KIR2DL2 absence	734	(48)	1		1		1	
KIR2DL2 presence	792	(52)	1.10 (0.91-1.32)	0.3	1.08 (0.94-1.25)	0.3	1.06 (0.86-1.32)	0.6
***KIR2DL1/3* in C1/C2 patients**								
KIR2DL1 clade 2 absence	839	(41)	1		1		1	
KIR2DL1 clade 2 presence	1185	(59)	1.11 (0.94-1.31)	0.2	1.12 (0.98-1.27)	0.09	1.13 (0.93-1.37)	0.2
KIR2DL3 clade 1 absence	794	(40)	1		1		1	
KIR2DL3 clade 1 presence	1189	(60)	1.14 (0.96-1.35)	0.14	1.08 (0.95-1.23)	0.2	1.02 (0.84-1.24)	0.8
***KIR3DL1*/HLA-B subtype combinations**								
Strong inhibiting *KIR3DL1*	1087	(26)	1		1		1	
Weak-inhibiting *KIR3DL1*	1106	(27)	1.10 (0.94-1.29)	0.2	1.08 (0.96-1.22)	0.2	1.05 (0.87-1.25)	0.6
Non-inhibiting *KIR3DL1*	1918	(47)	1.11 (0.96-1.27)	0.2	1.09 (0.98-1.21)	0.13	1.05 (0.89-1.24)	0.5
***KIR2DS1*/C1C2 epitope combinations**								
KIR2DS1 neg	2595	(62)	1		1		1	
KIR2DS1 pos / C1+	1355	(32)	1.03 (0.91-1.16)	0.7	1.00 (0.92-1.10)	0.9	0.98 (0.85-1.13)	0.7
KIR2DS1 pos / C2/C2	225	(5)	0.82 (0.62-1.07)	0.14	0.83 (0.68-1.02)	0.07	0.85 (0.63-1.15)	0.3
**KIR haplotype motif-based classification**								
Cen A/A	1994	(48)	1		1		1	
Cen A/B	1731	(42)	1.03 (0.91-1.16)	0.7	1.03 (0.94-1.12)	0.6	1.03 (0.89-1.18)	0.7
Cen B/B	413	(10)	0.89 (0.72-1.09)	0.3	0.90 (0.77-1.04)	0.2	0.90 (0.71-1.14)	0.4
Tel A/A	2518	(60)	1		1		1	
Tel A/B	1457	(35)	1.00 (0.88-1.12)	0.96	0.98 (0.90-1.07)	0.7	0.96 (0.83-1.10)	0.6
Tel B/B	200	(5)	0.98 (0.75-1.29)	0.9	1.02 (0.84-1.25)	0.8	1.08 (0.81-1.46)	0.6
Neutral (0 or 1 B-motif)	2907	(70)	1		1		1	
Better (≥2 B-motifs, no Cen B/B)	818	(20)	1.03 (0.90-1.19)	0.7	1.03 (0.92-1.14)	0.6	1.01 (0.85-1.20)	0.9
Best (≥2 B-motifs with Cen B/B)	413	(10)	0.88 (0.72-1.08)	0.2	0.89 (0.77-1.03)	0.13	0.89 (0.71-1.12)	0.3
**Sum inhibitory KIR - Ligands**								
Functional iKIR count (cont.)	4175	(100)	1.03 (0.97-1.10)	0.4	1.02 (0.97-1.07)	0.4	1.01 (0.93-1.09)	0.8
Inhibitory Score (cont.)	4175	(100)	1.03 (0.97-1.10)	0.3	1.02 (0.97-1.08)	0.4	1.01 (0.94-1.09)	0.8
**Net inhibitory / activating KIR – Ligands**								
Inhibitory (IM-)KIR Score (cont.)	4175	(100)	1.04 (0.94-1.15)	0.4	1.04 (0.97-1.12)	0.3	1.04 (0.93-1.17)	0.5
Weighted (w-)KIR Score (cont.)	4175	(100)	1.01 (0.90-1.12)	0.9	1.01 (0.93-1.09)	0.8	1.01 (0.89-1.15)	0.8
Missing-KIR-Score (cont.)	4175	(100)	0.95 (0.89-1.01)	0.09	0.96 (0.92-1.01)	0.11	0.98 (0.91-1.06)	0.6
Inhibitory-KIR-Score (cont.)	4175	(100)	1.06 (1.00-1.12)	0.044	1.05 (1.00-1.09)	0.036	1.03 (0.96-1.10)	0.4
Activating-KIR-Score (cont.)	4175	(100)	1.00 (0.93-1.07)	0.9	0.98 (0.93-1.03)	0.4	0.95 (0.87-1.03)	0.2
**Genotype signatures**								
G5 absence	3866	(93)	1		1		1	
G5 presence	309	(7)	1.09 (0.89-1.34)	0.4	1.02 (0.87-1.20)	0.8	0.93 (0.72-1.21)	0.6
G3 absence in Bw4 patients	2525	(95)	1		1		1	
G3 presence in Bw4 patients	133	(5)	0.96 (0.69-1.34)	0.8	1.01 (0.79-1.30)	0.9	1.08 (0.75-1.55)	0.7
G2 absence in C1/C1 patients	1346	(88)	1		1		1	
G2 presence in C1/C1 patients	180	(12)	1.00 (0.76-1.33)	0.99	0.90 (0.72-1.12)	0.3	0.76 (0.53-1.10)	0.14

Legend: TBI, total body irradiation; N, number; HR, hazard ratio; p, p-value; neg, negative; pos, positive; cen, centromeric; tel, telomeric; iKIR, inhibitory Killer cell Immunoglobulin like Receptors; cont, continuous; w-KIR-Score, weighted KIR-Score; IM-KIR-Score, inhibitory-missing KIR-ligand Score CIR, cumulative incidence of relapse; Hazard ratios were calculated in (cause-specific) multivariable Cox regression models stratified by registry (CIBMTR or EBMT), and adjusted for patient age, donor age, diagnosis, disease risk index, Karnofsky performance status, conditioning intensity, GvHD prophylaxis, sex match, CMV match, HLA-match, and stem cell source. The p-value of the Wald test is reported.

Table S6. Donor KIR genotype classifications for C1+ patients

Classifier	N	(%)	Relapse Incidence HR (95%-CI)	p	Event-free Survival HR (95%-CI)	p	Non-Relapse Mortality HR (95%-CI)	p
**KIR haplotype motif-based classification**								
Cen A/A	2055	(48)	1		1		1	
Cen A/B	1769	(42)	1.03 (0.92-1.16)	0.6	1.02 (0.93-1.12)	0.6	1.01 (0.88-1.16)	0.9
Cen B/B	424	(10)	0.88 (0.72-1.07)	0.2	0.92 (0.79-1.07)	0.3	0.97 (0.77-1.21)	0.8
Tel A/A	2575	(60)	1		1		1	
Tel A/B	1506	(35)	1.03 (0.92-1.16)	0.6	1.02 (0.93-1.11)	0.7	1.00 (0.87-1.15)	0.99
Tel B/B	205	(5)	1.09 (0.85-1.41)	0.5	1.05 (0.87-1.28)	0.6	1.01 (0.75-1.36)	0.96
Neutral (0 or 1 B-motif)	2971	(70)	1		1		1	
Better (≥2 B-motifs, no Cen B/B)	853	(20)	1.07 (0.93-1.23)	0.3	1.04 (0.94-1.16)	0.5	1.00 (0.85-1.17)	0.97
Best (≥2 B-motifs with Cen B/B)	424	(10)	0.88 (0.72-1.07)	0.2	0.92 (0.79-1.06)	0.2	0.96 (0.77-1.19)	0.7
**Sum inhibitory KIR – Ligands**								
Functional iKIR count (cont.)	4286	(100)	1.04 (0.97-1.10)	0.3	1.02 (0.97-1.06)	0.5	0.99 (0.92-1.07)	0.8
Inhibitory Score (cont.)	4286	(100)	1.03 (0.97-1.10)	0.3	1.01 (0.97-1.06)	0.6	0.99 (0.92-1.06)	0.8
**Net inhibitory / activating KIR – Ligands**								
Inhibitory (IM-)KIR Score (cont.)	4286	(100)	1.04 (0.95-1.14)	0.4	1.03 (0.96-1.11)	0.4	1.03 (0.92-1.14)	0.7
Weighted (w-)KIR Score (cont.)	4286	(100)	1.03 (0.92-1.15)	0.6	1.03 (0.94-1.11)	0.6	1.02 (0.90-1.16)	0.8
Missing-KIR-Score (cont.)	4286	(100)	0.97 (0.91-1.03)	0.3	0.98 (0.94-1.03)	0.5	1.00 (0.93-1.08)	0.95
Inhibitory-KIR-Score (cont.)	4286	(100)	1.04 (0.98-1.10)	0.2	1.03 (0.98-1.07)	0.2	1.01 (0.94-1.07)	0.8
Activating-KIR-Score (cont.)	4286	(100)	1.02 (0.95-1.09)	0.6	1.00 (0.95-1.05)	0.98	0.98 (0.90-1.06)	0.5

Legend: TBI, total body irradiation; N, number; HR, hazard ratio; p, p-value; cen, centromeric; tel, telomeric; iKIR, inhibitory Killer cell Immunoglobulin like Receptors; cont, continuous; w-KIR-Score, weighted KIR-Score; IM-KIR-Score, inhibitory-missing KIR-ligand Score CIR, cumulative incidence of relapse; Hazard ratios were calculated in (cause-specific) multivariable Cox regression models stratified by registry (CIBMTR or EBMT), and adjusted for patient age, donor age, diagnosis, disease risk index, Karnofsky performance status, conditioning intensity, GvHD prophylaxis, sex match, CMV match, HLA-match, and stem cell source. The p-value of the Wald test is reported.

Table S7. Donor KIR genotype classifications for C2/C2 patients

Classifier	N	(%)	Relapse Incidence		Event-free Survival		Non-Relapse Mortality	
			HR (95%-CI)	p	HR (95%-CI)	p	HR (95%-CI)	p
**KIR haplotype motif-based classification**								
Cen A/A	341	(47)	1		1		1	
Cen A/B	319	(44)	0.86 (0.64- 1.16)	0.3	0.86 (0.68- 1.08)	0.2	0.87 (0.60- 1.24)	0.4
Cen B/B	67	(9)	0.76 (0.45- 1.29)	0.3	0.76 (0.50- 1.14)	0.2	0.78 (0.41- 1.48)	0.4
Tel A/A	456	(62)	1		1		1	
Tel A/B	247	(34)	0.80 (0.59- 1.08)	0.14	0.85 (0.67- 1.07)	0.2	0.92 (0.64- 1.32)	0.7
Tel B/B	28	(4)	0.48 (0.19- 1.19)	0.11	0.66 (0.35- 1.23)	0.2	0.99 (0.42- 2.36)	0.99
Neutral (0 or 1 B-motif)	524	(72)	1		1		1	
Better (≥2 B-motifs, no Cen B/B)	136	(19)	0.82 (0.56- 1.20)	0.3	0.84 (0.63- 1.12)	0.2	0.88 (0.56- 1.37)	0.6
Best (≥2 B-motifs with Cen B/B)	67	(9)	0.78 (0.47- 1.31)	0.4	0.78 (0.52- 1.17)	0.2	0.81 (0.43- 1.52)	0.5
**Sum inhibitory KIR - Ligands**								
Functional iKIR count (cont.)	731	(100)	0.86 (0.68- 1.08)	0.2	0.90 (0.75- 1.07)	0.2	0.96 (0.73- 1.28)	0.8
Inhibitory Score (cont.)	731	(100)	0.86 (0.63- 1.16)	0.3	0.92 (0.73- 1.17)	0.5	1.02 (0.70- 1.50)	0.9
**Net inhibitory / activating KIR – Ligands**								
Inhibitory (IM-)KIR Score (cont.)	731	(100)	0.96 (0.74- 1.24)	0.7	0.96 (0.79- 1.17)	0.7	0.96 (0.70- 1.31)	0.8
Weighted (w-)KIR Score (cont.)	731	(100)	0.89 (0.69- 1.15)	0.4	0.90 (0.74- 1.09)	0.3	0.91 (0.66- 1.25)	0.6
Missing-KIR-Score (cont.)	731	(100)	0.90 (0.75- 1.09)	0.3	0.95 (0.82- 1.09)	0.50	1.02 (0.81- 1.28)	0.9
Inhibitory-KIR-Score (cont.)	731	(100)	1.14 (0.90- 1.44)	0.3	1.06 (0.88- 1.27)	0.6	0.94 (0.69- 1.26)	0.7
Activating-KIR-Score (cont.)	731	(100)	0.89 (0.77- 1.03)	0.13	0.88 (0.78- 0.99)	0.030	0.85 (0.71- 1.03)	0.10

Legend: TBI, total body irradiation; N, number; HR, hazard ratio; p, p-value; cen, centromeric; tel, telomeric; iKIR, inhibitory Killer cell Immunoglobulin like Receptors; cont, continuous; w-KIR-Score, weighted KIR-Score; IM-KIR-Score, inhibitory-missing KIR-ligand Score CIR, cumulative incidence of relapse; Hazard ratios were calculated in (cause-specific) multivariable Cox regression models stratified by registry (CIBMTR or EBMT), and adjusted for patient age, donor age, diagnosis, disease risk index, Karnofsky performance status, conditioning intensity, GvHD prophylaxis, sex match, CMV match, HLA-match, and stem cell source. The p-value of the Wald test is reported.

Table S8. Patient outcomes by presence of KIR genes

Classifier		N	(%)	Relapse Incidence HR (95%-CI)	p	Event-free Survival HR (95%-CI)	p	Non-Relapse Mortality HR (95%-CI)	p
KIR2DL1	presence	4846	(97)	1.07 (0.80-1.44)	0.6	1.10 (0.88-1.38)	0.4	1.15 (0.81-1.63)	0.4
	absence	171	(3)	1		1		1	
KIR2DL2	presence	2583	(51)	0.97 (0.88-1.08)	0.6	0.98 (0.90-1.05)	0.5	0.98 (0.87-1.10)	0.7
	absence	2434	(49)	1		1		1	
KIR2DL3	presence	4525	(90)	1.17 (0.98-1.40)	0.09	1.12 (0.98-1.28)	0.1	1.06 (0.87-1.30)	0.6
	absence	492	(10)	1		1		1	
KIR2DL5	presence	2420	(48)	1.00 (0.90-1.11)	0.98	0.97 (0.90-1.05)	0.5	0.94 (0.83-1.06)	0.3
	absence	2597	(52)	1		1		1	
KIR2DP1	presence	4865	(97)	1.01 (0.75-1.37)	0.9	1.09 (0.86-1.38)	0.5	1.21 (0.82-1.77)	0.3
	absence	152	(3)	1		1		1	
KIR2DS1	presence	1890	(38)	1.00 (0.90-1.11)	0.99	0.99 (0.91-1.07)	0.8	0.98 (0.86-1.10)	0.7
	absence	3127	(62)	1		1		1	
KIR2DS2	presence	2617	(52)	0.98 (0.88-1.08)	0.7	0.97 (0.90-1.05)	0.5	0.97 (0.86-1.09)	0.6
	absence	2400	(48)	1		1		1	
KIR2DS3	presence	1407	(28)	0.96 (0.86-1.08)	0.5	0.99 (0.91-1.08)	0.8	1.03 (0.90-1.18)	0.7
	absence	3610	(72)	1		1		1	
KIR2DS4	presence	4784	(95)	0.99 (0.77-1.26)	0.9	0.98 (0.82-1.18)	0.9	0.97 (0.74-1.28)	0.8
	absence	233	(5)	1		1		1	
full *KIR2DS4*	presence	1845	(37)	1.04 (0.93-1.15)	0.5	1.05 (0.97-1.14)	0.2	1.08 (0.96-1.22)	0.2
	absence	3172	(63)	1		1		1	
KIR2DS5	presence	1417	(28)	1.03 (0.92-1.16)	0.6	0.98 (0.90-1.07)	0.7	0.91 (0.80-1.05)	0.2
	absence	3600	(72)	1		1		1	
KIR3DL1	presence	4784	(95)	0.99 (0.77-1.26)	0.9	0.98 (0.82-1.18)	0.9	0.97 (0.74-1.28)	0.8
	absence	233	(5)	1		1		1	
KIR3DS1	presence	1853	(37)	1.01 (0.91-1.12)	0.9	0.99 (0.91-1.07)	0.8	0.96 (0.85-1.09)	0.5
	absence	3164	(63)	1		1		1	

Figure S1. Event-free survival, cumulative incidence of relapse and non-relapse mortality after transplantation from donors with homozygous centromeric or telomeric KIR gene motifs

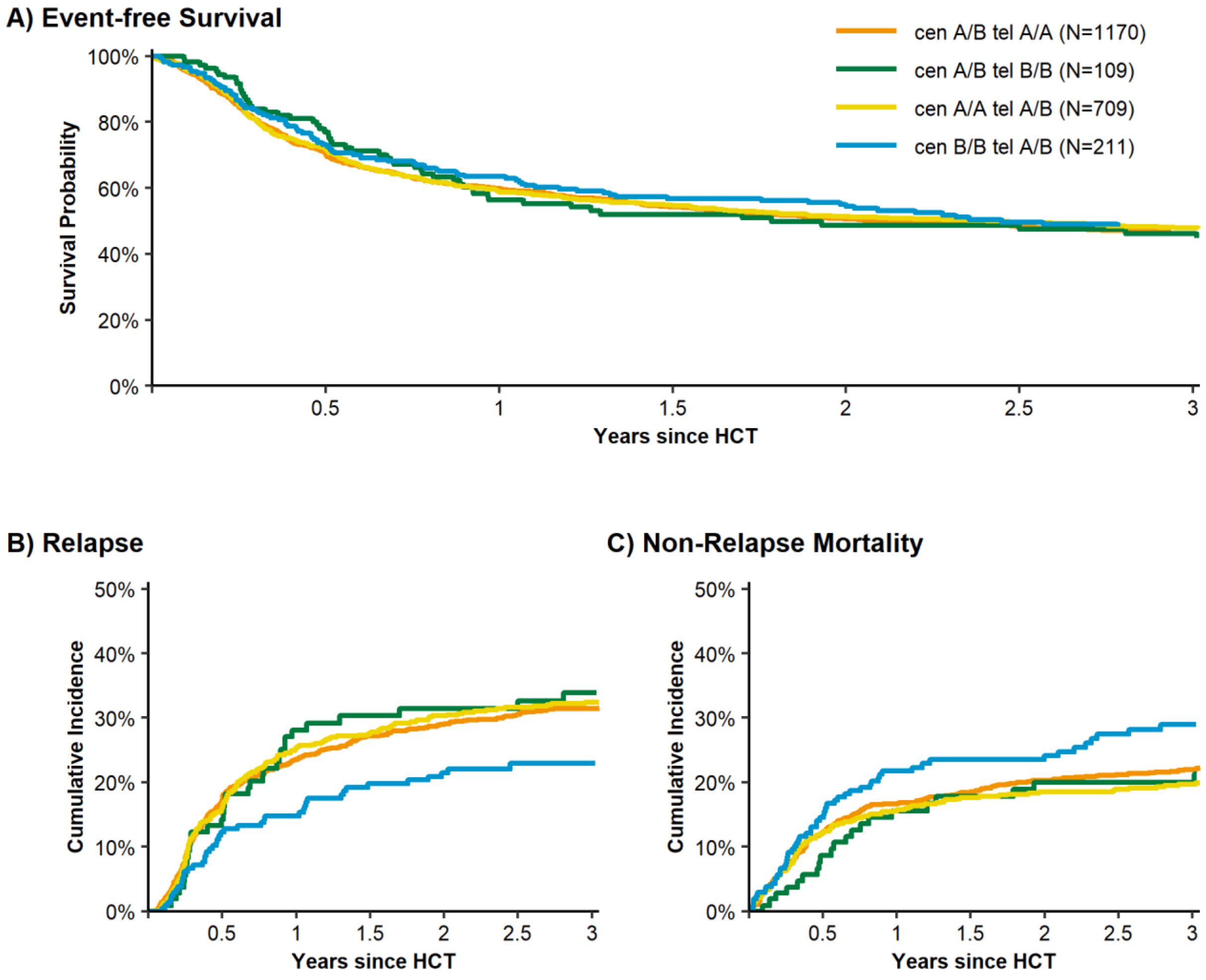

Panel A shows event-free survival from transplantation for patients with donors whose genotypes were homozygous for either the centromeric (cen) or the telomeric (tel) *KIR* gene motifs, but not both. Panels B and C show the cumulative incidences of relapse and non-relapse mortality, respectively.
